# Histone Arginine Methylation-Mediated Epigenetic Regulation of Discoidin Domain Receptor 2 Controls the Senescence of Human Bone Marrow Mesenchymal Stem Cells

**DOI:** 10.1155/2019/7670316

**Published:** 2019-07-11

**Authors:** Zhenyu Xu, Wenming Wu, Fang Shen, Yue Yu, Yue Wang, Charlie Xiang

**Affiliations:** ^1^State Key Laboratory for Diagnosis and Treatment of Infectious Diseases and Collaborative Innovation Center for Diagnosis and Treatment of Infectious Diseases, The First Affiliated Hospital, School of Medicine, Zhejiang University, Hangzhou 310027, China; ^2^Department of Orthopedics, Beijing Ditan Hospital, Capital Medical University, Beijing 100015, China; ^3^Department of Orthopaedic Surgery's Spine Division, The Affiliated Hospital of Medical School of Ningbo University, Ningbo, 315020, China; ^4^Department of Thyroid and Breast, Changhai Hospital, Shanghai 200433, China; ^5^Department of Histology and Embryology, Second Military Medical University, Shanghai 200433, China; ^6^Shanghai Key Laboratory of Cell Engineering, China

## Abstract

The application of human bone marrow mesenchymal stem cells (hBM-MSCs) in cell-based clinical therapies is hindered by the limited number of cells remaining after the initial isolation process and by cellular senescence following *in vitro* expansion. Understanding the process of *in vitro* senescence in hBM-MSCs would enable the development of strategies to maintain their vitality after cell culture. Herein, we compared the gene expression profiles of human embryonic stem cells and human BM-MSCs from donors of different ages. We first found that the expression of discoidin domain receptor 2 (DDR2) in adult donor-derived hBM-MSCs was lower than it was in the young donor-derived hBM-MSCs. Moreover, *in vitro* cultured late-passage hBM-MSCs showed significant downregulation of DDR2 compared to their early-passage counterparts, and siRNA inhibition of DDR2 expression recapitulated features of senescence in early-passage hBM-MSCs. Further, we found through knockdown and overexpression approaches that coactivator-associated arginine methyltransferase 1 (CARM1) regulated the expression level of DDR2 and the senescence of hBM-MSCs. Finally, chromatin immunoprecipitation analysis confirmed direct binding of CARM1 to the DDR2 promoter region with a high level of H3R17 methylation in early-passage hBM-MSCs, and inhibition of CARM1-mediated histone arginine methylation decreased DDR2 expression and led to cellular senescence. Taken together, our findings suggest that DDR2 plays a major role in regulating the *in vitro* senescence of hBM-MSCs and that CARM1-mediated histone H3 methylation might be the upstream regulatory mechanism controlling this function of DDR2.

## 1. Introduction

Mesenchymal stem cells (MSCs) are multipotent adult stem cells with self-renewal capacity, multilineage differentiation potential, and immunomodulatory properties [1]. MSCs have been considered a promising candidate for cell-based clinical therapies for over a decade [2]. Although MSC-like cell populations have been isolated from many types of tissues (e.g., adipose tissue [3] and umbilical cord [4]), human bone marrow- (BM-) derived MSCs (hBM-MSCs) are the best-characterized adult stem cells and represent the major source of MSCs for clinical applications. Due to the invasive nature of bone marrow trephine, however, *in vivo* collection of hBM-MSCs usually results in a limited cell yield. Thus, to harvest high quantities of hBM-MSCs, *in vitro* cell expansion by long-term culture is required [5]. Unfortunately, *in vitro* culture has been shown to alter the capacity of MSCs to differentiate into various types of tissue [6]. For example, the “adipogenic switch,” or the loss of osteogenic potential and gain of adipogenic potential, has been observed in MSCs at advanced ages [7, 8]. More importantly, hBM-MSCs in late passages have been shown to become senescent [9]. Therefore, efforts have been made to unveil the mechanisms underlying the *in vitro* senescence of hBM-MSCs to expand the potential for the use of hBM-MSCs in clinical applications [10–13]. On the other hand, young donor-derived hBM-MSCs have different proliferative abilities and senescence characteristics during the *in vitro* passaging process compared to adult hBM-MSCs [14–16]. Therefore, the *in vitro* senescence potential of young donor-derived hBM-MSCs lies somewhere between those of human embryonic stem cells (hESCs) and hBM-MSCs.

Discoidin domain receptor 2 (DDR2) has recently been shown to play an essential role in skeletal development and the differentiation of marrow progenitor cells to osteoblasts while suppressing marrow adipogenesis [17]. In the present study, DDR2 was first identified as differentially expressed among hBM-MSCs with different senescence characteristics. This association of DDR2 with hBM-MSC cellular senescence was confirmed by the decreased DDR2 expression we observed in the late-passage hBM-MSCs and the recapitulation of senescence features we observed in early-passage hBM-MSCs following siRNA inhibition of total and phosphorylated DDR2 expression. Previous studies have shown that hMSCs acquire specific epigenetic changes during *ex vivo* expansion [18, 19] and that those DNA methylations are associated with the promoter regions of genes involved in cell differentiation [20]. Our previous study showed that coactivator-associated arginine methyltransferase 1 (CARM1) plays a key role in hESC resistance to differentiation by regulating the expression of pluripotency genes via CARM1-mediated histone H3 methylation [21]. In the present study, we discovered that CARM1 upregulates both total and phosphorylated DDR2 expression in hBM-MSCs via increased methylation of histone H3 in the DDR2 promoter region and can contribute to the rejuvenation of late-passage hBM-MSCs.

## 2. Materials and Methods

### 2.1. Cell Culture

Human bone marrows were obtained from the Changhai Hospital, Shanghai, China, following written informed consent of the patients regarding their participation in the study. The study protocol was approved by the Ethics Committee and Science Committee of the Changhai Hospital. hBM-MSCs were isolated and cultured as follows: A total of 3 mL of Ficoll-Paque media (GE Healthcare) was added to the centrifuge tube, and 4 mL of diluted blood sample was subsequently layered onto the Ficoll-Paque media solution. Following centrifugation at 400 ×*g* for 30 min at 18°C, the upper layer containing plasma and platelets was discarded, and the interface layer containing mononuclear cells was transferred carefully to a new tube and mixed with three volumes of 1x phosphate-buffered saline (PBS). The centrifugation process was repeated for two additional times, and the resulting cell pellets were collected and resuspended in appropriate culture media (DMEM containing 10% FBS, 100 IU/mL streptomycin and penicillin, and 2 mM L-glutamine, all from GIBCO Invitrogen). At 80% confluence, the cells were trypsinized with 0.05% trypsin and 0.025% ethylenediaminetetraacetic acid (EDTA). Primary hBM-MSC cell lines were partially cryopreserved following each passage for further studies. The identifications of hBM-MSCs were carried out according to the previous study conducted by our group, and^22^ the results will be presented in subsequent reports. Ellagic acid (100 mM) was added to the medium for a site-specific inhibition of CARM1. Unless otherwise specified, early-passage hBM-MSCs corresponded to cells with a passage number lower than 3, while late-passage hBM-MSCs corresponded to cells with a passage number higher than 7.

### 2.2. Differential Gene Expression (DGE) Analysis

Human BM-MSCs that were derived from young donors (from less than 30-year-old healthy donors, Y-hBM-MSCs) were subjected to gene expression profile analysis (Affymetrix GeneChip Human U133) by Genminix Informatics Ltd. Co. (Beijing). Global DGE analysis was conducted by cluster analysis (unsupervised classification) of the microarray data of Y-hBM-MSCs compared with those of adult hBM-MSCs (from more than 50-year-old healthy donors) and hESCs (from H1 cell lines). The selection criteria for differentially expressed genes were set as the genes with at least 3-fold change in their expression level among these three types of cells.

### 2.3. Growth Curve, Cell Viability, and Colony-Forming Unit Fibroblast (CFU-F) Assay

The cells were seeded to 48-well plates at the density of 5 × 10^3^ cells per well and then digested and counted by cytometry at 6, 12, 24, 36, 48, 72, and 96 h time points for growth curve analysis. Cell viability was measured by addition of 10 *μ*L of cell counting kit-8 (CCK-8) solution to each well and subsequent incubation of the cells for 1 to 4 h. The absorbance was monitored spectrophotometrically at 450 nm using a microplate reader. A CFU-F assay was conducted, by addition of cells to a T25 flask at a density of 1 × 10^5^ cells. Following one week of cell culture, the cells were washed by 1x PBS and fixed with methanol for 5 min. The methanol was decanted, and the cells were air-dried and stained with 5% Giemsa solution for 5 min at room temperature. The cells were subsequently washed 2 times with deionized water, air-dried, and observed under the microscope. Cell aggregates with a diameter of 1-8 mm were included in the analysis, whereas more than 20 cells were counted as one CFU-F colony.

### 2.4. 5-Ethynyl-2′-deoxyuridine (EdU) Incorporation Assays

EdU incorporation assays were carried out as follows: The cells were plated in 48-well plates and left to grow. At 80% confluence, 50 *μ*L of EdU was added to the culture medium, and the cells were incubated at 37°C for 4 h. The cells were finally washed twice with 1x PBS and fixed in 4% polyformaldehyde for 30 min. Subsequently, the cells were stained with Apollo 567 and DAPI solution according to the instructions provided by the manufacturer.

### 2.5. Real-Time PCR

Total RNA was extracted using TRIzol (Invitrogen). The total RNA was subjected to reverse transcription with specific primers. Briefly, the first-strand complementary DNA (cDNA) was synthesized by reverse transcription of the mRNA with random primers and oligo dT. cDNAs were amplified by real-time quantitative PCR (qPCR) using the Power SYBR Green PCR Master Mix (Roche) in a StepOnePlus System (Applied Biosystems) according to the standard protocol.

### 2.6. *β*-Galactosidase Staining

A *β*-Galactosidase (*β*-Gal) staining kit was purchased from Cell Signaling Technology (CST, #9860), and the experiment was carried out according to the instructions provided by the manufacturer. The culture medium was removed, and the cells were washed with 1x PBS. A total of 1 mL 1x fixative solution was added to each 35 mm well, and the cells were fixed for 10 to 15 min at room temperature. The plate was rinsed with 1x PBS two times, and 1 mL *β*-Galactosidase staining solution was added to each 35 mm well. Following addition of the Galactosidase staining solution, the plate was incubated at 37°C overnight in a dry incubator in the absence of CO_2_, and the cells were observed under a microscope for the development of blue color.

### 2.7. Immunofluorescence Staining

Following removal of the culture medium, the cells were first fixed in 2% paraformaldehyde for 15 min at room temperature and permeabilized with 0.1% Triton X-100 for 15 min. Following blocking with 2% BSA for 45 min, primary antibody (anti-H2A.X phospho S139, 1 : 200, CST) was added, and the cells were incubated overnight. The incubation time of the secondary antibody (1 : 1000, CST) was 30 min, whereas the incubation in 0.1% DAPI was 5 min. The results were observed under an inverted fluorescence microscope.

### 2.8. siRNA Transfection

siRNAs that were designed to specifically target CARM1 and DDR2 were synthesized by GenePharma (Shanghai, China). Lipofectamine RNAiMAX (Invitrogen) was used in this study. Briefly, siRNA and the RNAiMAX transfection reagent were initially diluted with serum-free culture medium and gently mixed by pipetting. The mixed reagent was incubated for 5 min at room temperature and subsequently added to the cell culture medium. The cells were further cultured at 37°C in 5% CO_2_ for 24 to 48 h (for mRNA analysis) and/or 48 to 96 h (for protein analysis).

### 2.9. Plasmid Construction

EcoRI and KpnI were selected as the restriction endonucleases. The DDR2 gene was amplified from hBM-MSC cDNA and cloned into a pcDNA3.1-flag plasmid vector. CARM1 overexpression vectors were constructed as described in previous studies.^21^

### 2.10. Western Blot Analysis

Total protein concentration was determined with the BCA Protein Assay Kit (Beyotime). The protein extracts were separated by 10% SDS-PAGE and transferred to polyvinylidene difluoride membranes (Millipore). Nonspecific protein binding was achieved by incubation of the membranes with 5% dried skim milk for 1 h, and the membranes were incubated overnight with antibodies against CARM1 (1 : 1000, Abcam), histone H3R17 dimethylation (1 : 800 Millipore), p15 (1 : 1000, Abcam), p16 (1 : 1000, Abcam), p21 (1 : 800, Abcam), TERT (1 : 500, Abcam), DDR2 (1 : 500, R&D), phospho-DDR2 Y740 (1 : 500, R&D), and *β*-actin (1 : 1000, ABclonal). The next day, the membranes were incubated with horseradish peroxidase-conjugated secondary antibodies (1 : 5000, ABGENT) for 1 h. The results were visualised by the Chemiluminescent HRP Substrate (Millipore) and recorded by a Bioshine ChemiQ imaging scanner (Bioshine).

### 2.11. Comet Assay

Cell pellets were collected by centrifugation and washed once in cold 1x PBS. Following resuspension at a density of 1 × 10^5^ cells/mL in cold PBS, the cells were combined with low melting point (LMP) agarose (at 37°C) at a ratio of 1 : 10 (*v*/*v*). A total of 75 *μ*L of the resultant solution was pipetted immediately on comet slides. The slides were initially placed at a horizontal position at 4°C in the dark for 10 min and immersed in prechilled lysis solution. The samples were incubated at 4°C for 30 min. Subsequently, the slides were immersed in alkaline solution (pH > 1) for 30 min at room temperature in the dark. Following washing in 1x TBE buffer for 5 min (two times), the slides were transferred to a horizontal electrophoresis apparatus, and electrophoresis was conducted at a voltage of 10 min. The slides were subsequently fixed in 70% ethanol for 5 min and air-dried. The comet tail was scored according to the DNA content (intensity). At least 100 cells were scored per sample slide.

### 2.12. Chromatin Immunoprecipitation (ChIP) Assay

ChIP assays were conducted using the EZ-Magna ChIP™ A/G Chromatin Immunoprecipitation Kit (Millipore). Briefly, chromatin was immunoprecipitated with anti-CARM1 (Abcam) and anti-histone H3R17di-me (Abcam). Anti-RNA polymerase (Millipore) was used as a positive control, and anti-IgG (Millipore) was used as a negative control. ChIP-derived DNA was quantified using real-time PCR with SYBR Green (Applied Biosystems). A total of 4 pairs of primers were designed for the promoter region of each gene in order to detect the enriched genomic DNA fragments. The qPCR values were normalised according to the values of a promoter region of GAPDH. The normalised input signal was defined as 1. The fold enrichment value is indicated as the normalised ChIP signal divided by the normalised input signal.

### 2.13. Statistical Analysis

The data are presented as the mean ± SD. The comparisons of the differences between groups regarding protein, gene, and mRNA levels as well as immunofluorescence-positive cell numbers and fold enrichments were determined by one-way or two-way repeated measures analysis of variance (ANOVA), followed by Dunnett's post hoc test. The comparison of the samples with regard to the CCK8 absorbance was carried out with unpaired Student's *t*-test. A *P* value of less than 0.05 (*P* < 0.05) was considered as statistically significant. The graphical and data analyses were carried out with SPSS v.18.0 (IBM Corp., Armonk, NY, USA).

## 3. Results

### 3.1. hBM-MSCs Showed Phenotypical Changes in Cellular Senescence after Prolonged *In Vitro* Culture

**Figure 1:**
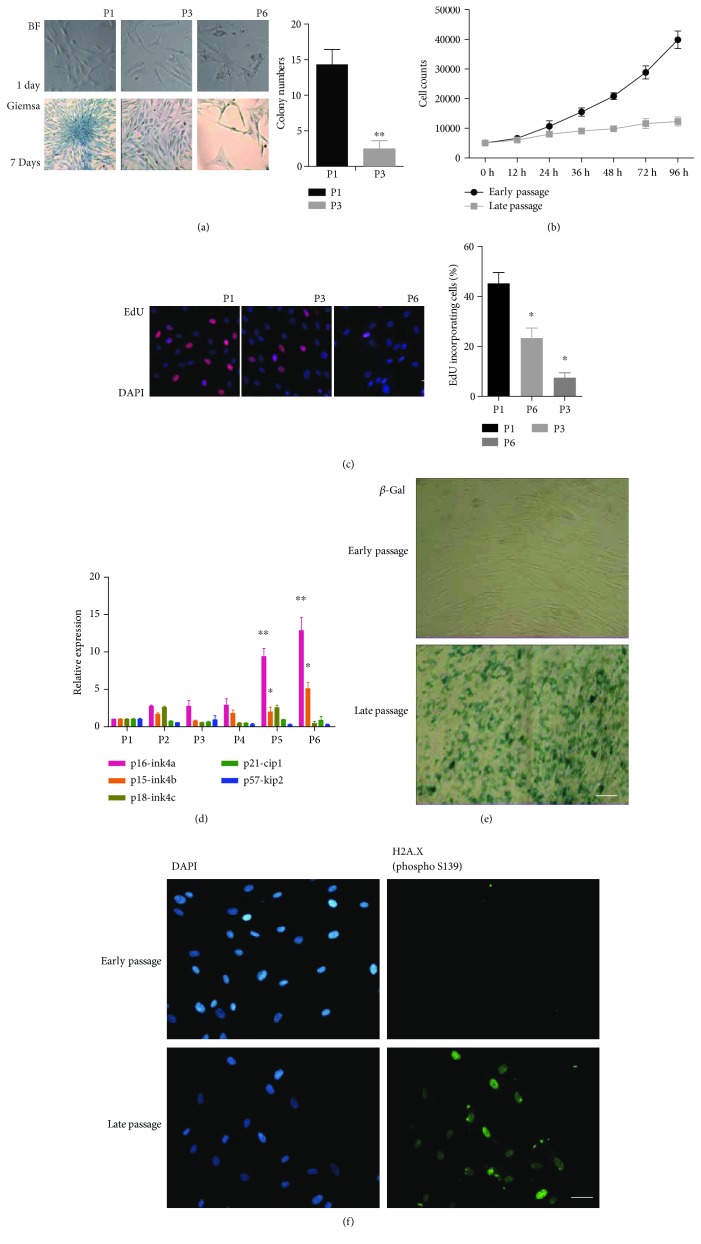
hBM-MSCs exhibited phenotypical changes associated with cellular senescence after prolonged *in vitro* culture. (a) Compared with early-passage cells, the late-passage hBM-MSCs showed elongated cellular processes, reduced clone number, and increased cell area. (b) The growth curve showed that the doubling time of late-passage cells was extended compared to that of early-passage cells. (c) EdU cell proliferation assay showed a marked reduction in EdU-positive cell nuclei among the late-passage cells, suggesting a decrease in cell proliferation capacity. (d) qPCR analysis of the cyclin-dependent kinase (CDK) inhibitors (CKI) demonstrated a significant increase in p16 and p15 expression levels in the late-passage cells (passage 7). (e) The expression of the senescence-associated marker *β*-Gal was also markedly increased in late-passage cells. (f) Immunofluorescence staining showed increased endogenous levels of histone H2A.X protein with Ser139 phosphorylation (H2A.X Ser139) in late-passage cells. Bars represent the standard error of the mean ± S.D. from three repeats. Statistical significance was determined by ANOVA and Student's *t*-test. ^∗∗^*P* < 0.01, *n* = 6.

Compared to their early-passage counterparts, the late-passage hBM-MSCs exhibited elongated cellular processes, reduced clone number, and increased surface area (Figure 1(a)). The growth curve showed that the doubling time for the late-passage cells was extended compared to that of early-passage cells (Figure 1(b)). The EdU cell proliferation assay showed a marked reduction in EdU-positive cell nuclei in the late-passage cells, suggesting a decrease in cell proliferation capacity (Figure 1(c)). Analysis of the cyclin-dependent kinase (CDK) inhibitors (CKI) by qPCR demonstrated a significant increase in p16 and p15 expression in the late-passage cells (Figure 1(d)). Expression of the senescence-associated marker *β*-Gal was also markedly increased in these late-passage cells (Figure 1(e)). Immunofluorescence staining showed that the endogenous levels of histone H2A.X protein phosphorylated at the Ser139 residue (H2A.X Ser139) were also increased in the late-passage cells (Figure 1(f)).

### 3.2. DDR2 Was Differentially Expressed in Young Donor-Derived hBM-MSCs Compared to Adult Donor-Derived hBM-MSCs

**Figure 2:**
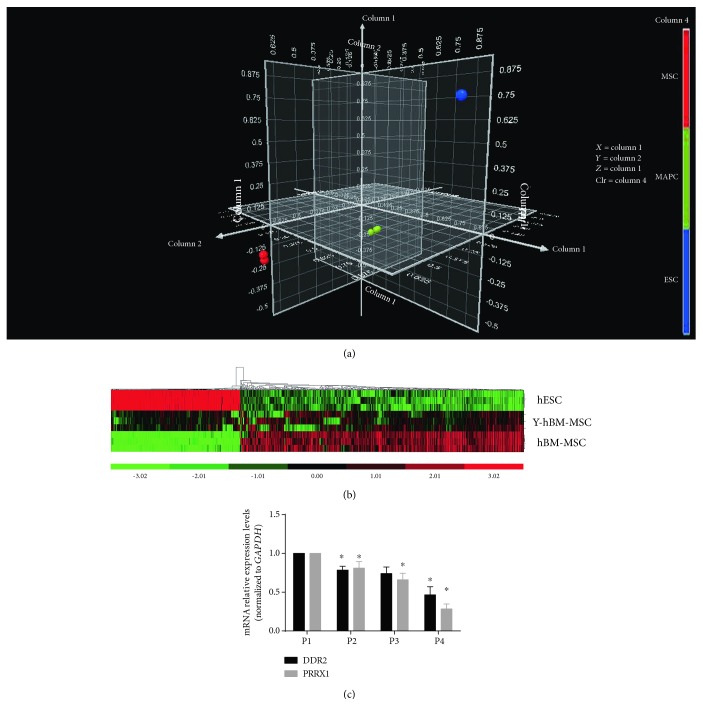
DDR2 was differentially expressed in young adult-derived hBM-MSCs compared to hESCs and hBM-MSCs. (a) Microarray gene expression profiling of young adult-derived hBM-MSCs (Y-hBM-MSCs) and subsequent comparisons with hBM-MSC and hESC GEO data revealed a higher correlation between the gene expression profiles of Y-hBM-MSCs and hBM-MSCs than between Y-hBM-MSCs and hESCs. (b) Bioinformatics analysis identified genes that were either sequentially downregulated or upregulated from hESCs, Y-hBM-MSCs, and hBM-MSCs with at least a 3-fold difference between the adjacent groups. (c) qPCR analysis of *in vitro* cultured hBM-MSC gene expression further verified the downregulation of two genes, DDR2 and PPRX1, with the increasing cell passage number. P: passage. Bars represent the standard error of the mean ± S.D. from three repeats. Statistical significance was determined by ANOVA and Student's *t*-test. ^∗∗^*P* < 0.01, *n* = 6.

To identify genes playing key roles in the regulation of cellular proliferation and senescence, the gene expression profiles of young donor-derived hBM-MSCs (Y-hBM-MSCs) were examined by microarray analysis and compared with those of adult donor-derived hBM-MSCs and hESCs retrieved from the GEO database. The cluster analysis revealed a higher correlation between the gene expression profiles of Y-hBM-MSCs and hBM-MSCs than those of Y-hBM-MSCs and hESCs (Figure 2(a)). Bioinformatics analysis identified the differentially expressed gene sets that were sequentially changed in hESCs, Y-hBM-MSCs, and hBM-MSCs (with at least a 3-fold difference between adjacent groups) (Figures 2(b) and 2(c)). Among them, a group of cell cycle or cell proliferation regulating genes, including DDR2 and PPRX1, were identified by applying cluster enrichment analysis to Gene Ontology (data not shown). *In vitro* qPCR analysis of DDR2 and PPRX1 expression in hBM-MSCs also confirmed their downregulation with an increasing cell passage number (Figure 2(c)).

### 3.3. Inhibition of DDR2 Induced *In Vitro* Cellular Senescence and DNA Damage in hBM-MSCs

**Figure 3:**
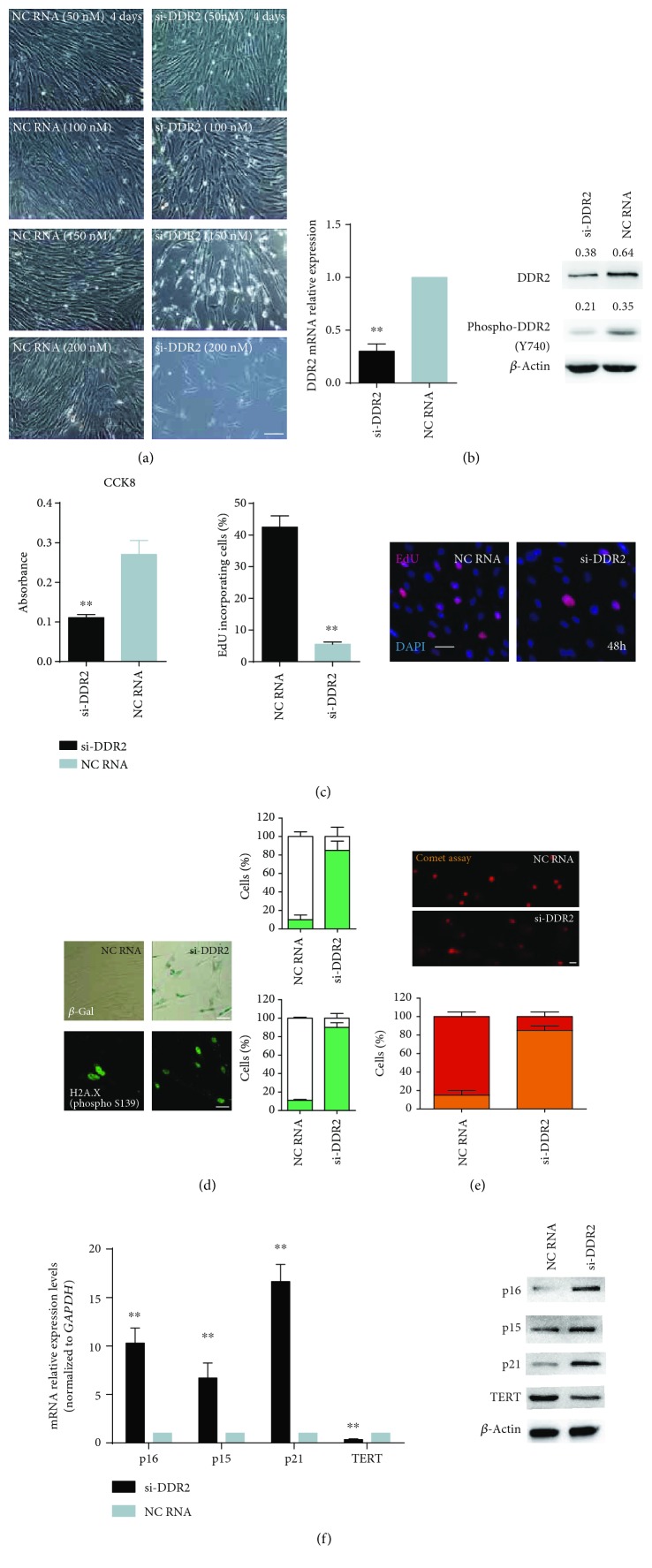
Inhibition of DDR2 induced *in vitro* cellular senescence and DNA damage in hBM-MSCs. (a) hBM-MSCs treated for four days with siRNA-targeted DDR2 (si-DDR2) exhibited reduced clone number and morphological changes characteristic of late-passage senescent cells. (b) qPCR and Western blot analyses confirmed that the mRNA, total protein, and phosphorylated protein levels of DDR2 were significantly reduced following DDR2 knockdown. (c) Both CCK8 and EdU assays indicated decreased cellular proliferation capacity after DDR2 knockdown. (d) The senescence marker *β*-Gal and DNA damage marker H2A.X Ser139 were increased in si-DDR2-transfected cells. (e) Comet assay showed that a much higher percentage of si-DDR2-transfected hBM-MSCs exhibited comet tails compared to the control. (f) DDR2 knockdown significantly increased p15, p21, and p16 expression and reduced TERT expression both at the mRNA and protein levels. For WB assays, the relative expression of phosphorylated DDR2 and total DDR2 refers to *β*-actin that was determined using density analysis, and the ratios were shown above the bands. Bars represent the standard error of the mean ± S.D. from three repeats. Statistical significance was determined by ANOVA and Student's *t*-test. ^∗∗^*P* < 0.01, *n* = 6.

After hBM-MSCs were treated for four days with siRNA targeted against DDR2, the transfected cells exhibited reduced clone number and morphological changes characteristic of late-passage senescent cells (Figure 3(a)). The maximum efficacy was observed at an siRNA concentration of 200 nM. qPCR and Western blot analyses confirmed that DDR2 mRNA, total protein, and phosphorylated protein levels were significantly reduced following treatment with 150 nM siRNA (Figure 3(b)). Both CCK8 and EdU assays indicated decreased cellular proliferation capacity after siRNA administration (Figure 3(c)). As suggested by the increased percentages of positive cells, siRNA-transfected cells also exhibited a marked increase in the expression of the senescence marker *β*-Gal and the DNA damage marker H2A.X Ser139 (Figure 3(d)). Increased DNA damage in these cells was further verified by the comet assay, which showed that a much higher percentage (83%) of transfected hBM-MSCs exhibited comet tails compared to controls (Figure 3(e)). Inhibition of DDR2 expression by siRNA also significantly increased p15, p21, and p16 mRNA levels and decreased the amount of TERT mRNA (Figure 3(f)). Western blot analysis showed that the protein levels of p15, p21, and p16 also increased after DDR2 interference, whereas the protein level of TERT decreased (Figure 3(f)).

### 3.4. CARM1 Was the Upstream Regulator of DDR2 in hBM-MSC Resistance against Senescence

**Figure 4:**
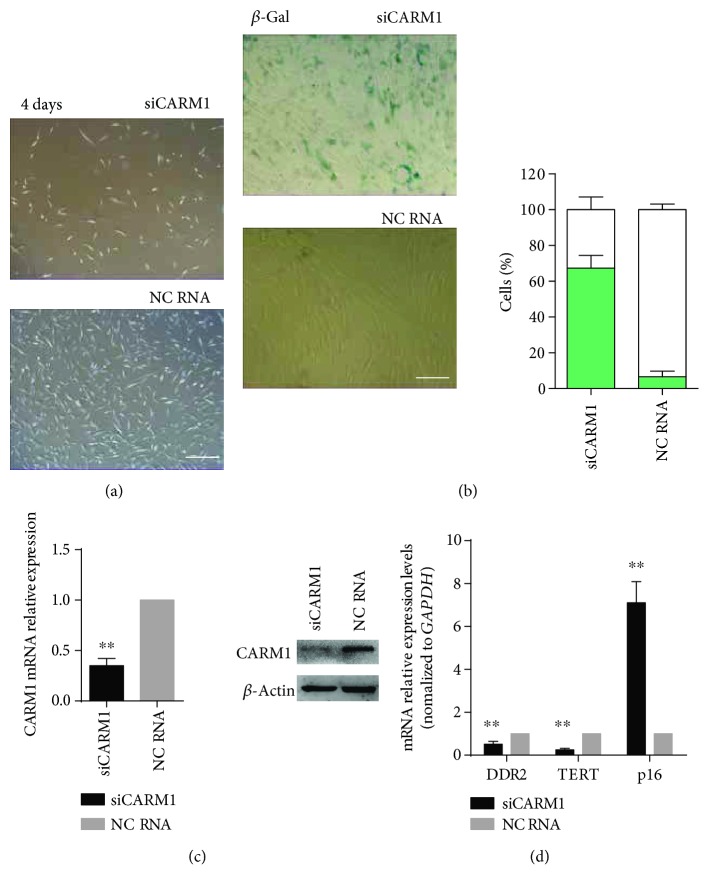
Inhibition of CARM1 expression in hBM-MSCs induced cellular senescence and DDR2 downregulation. (a) Early-passage hBM-MSCs treated for four days with siRNA targeted against CARM1 exhibited reduced clone number and morphological changes characteristic of late-passage senescent cells. (b) After 1 week of *in vitro* culture, the percentage of cells positive for the senescence marker *β*-Gal increased significantly. (c) qPCR and Western blot analyses confirmed that CARM1 mRNA and protein levels were significantly reduced in these cells following siRNA administration. (d) The mRNA level of p16 was also markedly increased in siRNA-transfected cells, while the mRNA levels of TERT and DDR2 were significantly decreased. Bars represent the standard error of the mean ± S.D. from three repeats. Statistical significance was determined by ANOVA and Student's *t*-test. ^∗∗^*P* < 0.01, *n* = 6.

After early-passage hBM-MSCs were treated for four days with siRNA targeted against CARM1, the transfected cells demonstrated reduced clone number and morphological changes characteristic of late-passage senescent cells (Figure 4(a)). After one week of culture, the percentage of cells positive for the senescence marker *β*-Gal increased significantly (Figure 4(b)). qPCR and Western blot analyses confirmed that CARM1 mRNA and protein levels were significantly reduced after siRNA administration (Figure 4(c)). The mRNA level of p16 was markedly increased in the siRNA-transfected cells, while TERT and DDR2 mRNA levels were significantly decreased (Figure 4(d)).

**Figure 5:**
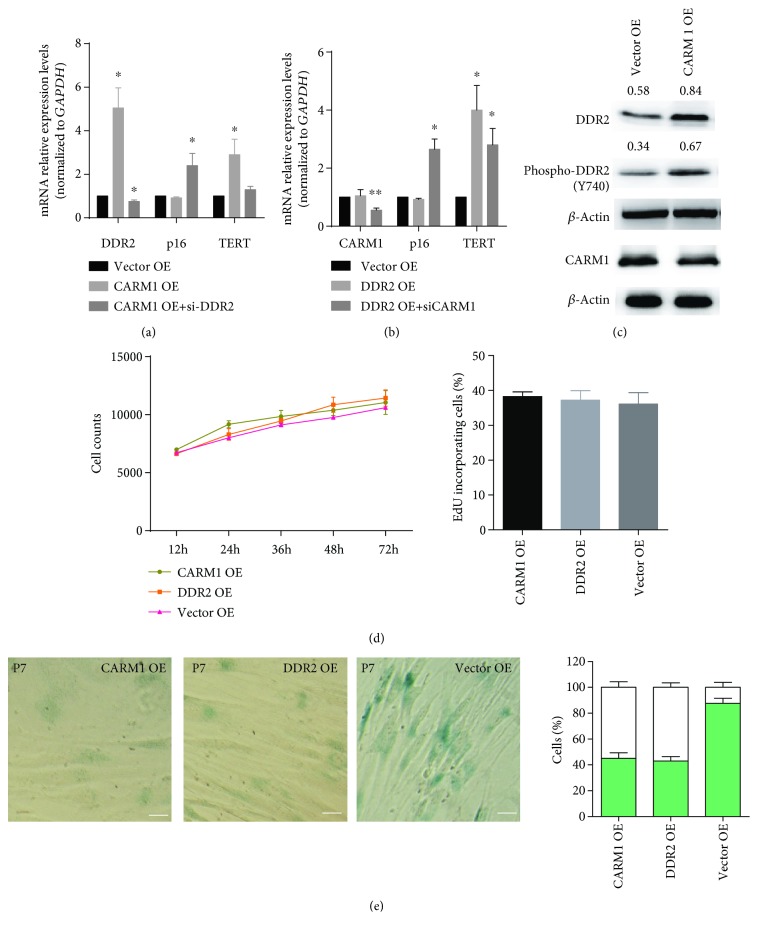
Overexpression of CARM1 led to senescence resistance through DDR2. (a) Overexpression of CARM1 led to a significant upregulation in both DDR2 and TERT expressions but had no effect on p16 expression. Simultaneous inhibition of DDR2 with CARM1 overexpression increased the mRNA level of p16. (b) Although overexpression of DDR2 alone had no effect on the expression of CARM1 or p16, simultaneous inhibition of CARM1 with DDR2 overexpression significantly increased the mRNA level of p16. (c) Western blot analysis confirmed that overexpression of CARM1 increased the protein and phosphorylation level of DDR2, whereas overexpression of DDR2 had no effect on the protein level of CARM1. (d) However, overexpression of either CARM1 or DDR2 in hBM-MSCs altered their proliferation rate, as determined by growth curve and EdU incorporation assays. (e) In contrast, when CARM1 or DDR2 was overexpressed, the percentage of cells positive for the senescence marker *β*-Gal decreased significantly after 1 week of *in vitro* culture. For WB assays, the relative expression of phosphorylated DDR2 and total DDR2 refers to *β*-actin that was determined using density analysis, and the ratios were shown above the bands. Bars represent the standard error of the mean ± S.D. from three repeats. Statistical significance was determined by ANOVA and Student's *t*-test. ^∗∗^*P* < 0.01, *n* = 6.

To examine whether CARM1 regulates cellular senescence through its action on DDR2, CARM1 was first overexpressed in hBM-MSCs. This overexpression led to significant upregulation of DDR2 and TERT expression, whereas it had no effect on p16 expression (Figure 5(a)). However, simultaneous inhibition of DDR2 by siRNA and overexpression of CARM1 led to increased p16 expression (Figure 5(a)). In contrast, DDR2 overexpression did not affect the expression of CARM1 or p16 (Figure 5(b)). On the other hand, simultaneous inhibition of CARM1 by siRNA and overexpression of DDR2 significantly increased p16 expression (Figure 5(b)). Western blot analysis confirmed that CARM1 overexpression increased the total and phosphorylated protein levels of DDR2, while DDR2 overexpression had no effect on the protein level of CARM1 (Figure 5(c)). Interestingly, neither CARM1 nor DDR2 overexpression altered the proliferation of hBM-MSCs (Figure 5(d)). However, after culturing the CARM1- or DDR2-overexpressing cells for one week (passage 7), the percentage of cells positive for the senescence marker *β*-Gal decreased significantly (Figure 5(e)).

**Figure 6:**
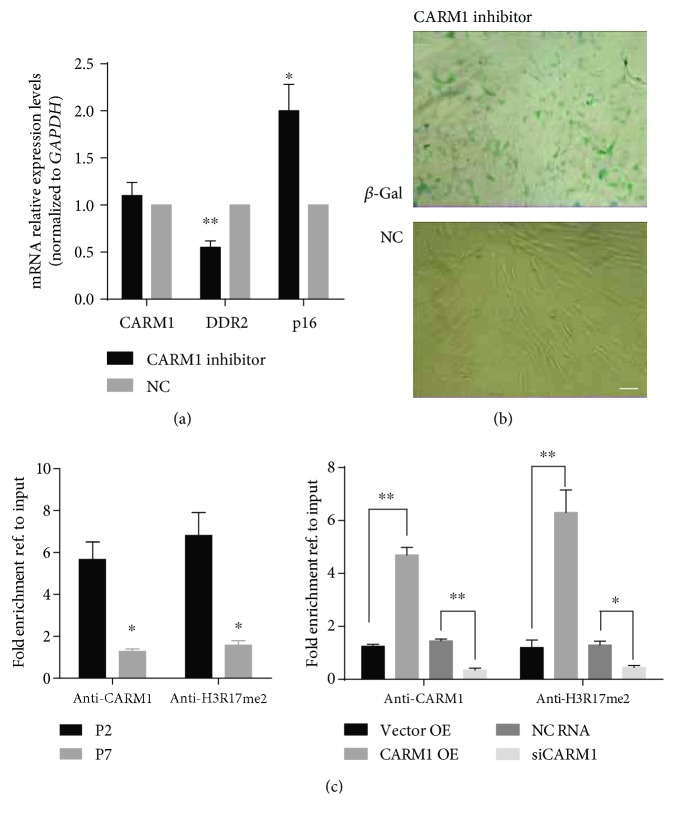
CARM1 regulated DDR2 expression through catalyzing H3R17 methylation in the DDR2 promoter region. (a) Specific inhibition of CARM1-mediated histone arginine methylation was achieved by treatment with 100 *μ*M ellagic acid. CARM1 inhibition decreased DDR2 expression and increased p16 expression and led to the generation of a significant number of senescent cells. (b) ChIP results verified that CARM1 was capable of binding to the promoter region of DDR2 and preferentially methylated H3R17. In early-passage (P2) hBM-MSCs, clear coincidental CARM1 accumulation and H3R17 methylation on the DDR2 promoter region were identified, while no similar results were found in late-passage (P7) cells. (c) hBM-MSCs with CARM1 overexpression also showed similar coincidental CARM1 accumulation and H3R17 methylation, while those with CARM1 inhibition exhibited a significant decrease in CARM1 accumulation and H3R17 methylation. Bars represent the standard error of the mean ± S.D. from three repeats. Statistical significance was determined by ANOVA and Student's *t*-test. ^∗∗^*P* < 0.01, *n* = 6.

Specific inhibition of CARM1-mediated histone arginine methylation in hBM-MSCs was achieved by treatment with 100 *μ*M ellagic acid. CARM1 inhibitor treatment led to decreased DDR2 expression and increased p16 expression, as well as the generation of a significant number of senescent cells (Figure 6(a)). ChIP results verified that CARM1 was capable of binding to the promoter region of DDR2 and preferentially methylated H3R17. A clear coincidental CARM1 accumulation and H3R17 methylation on this promoter region were identified in early-passage (passage number = 2) but not late-passage (passage number = 7) hBM-MSCs (Figure 6(b)). hBM-MSCs with CARM1 overexpression also showed similar coincidental CARM1 accumulation and H3R17 methylation, while those with CARM1 inhibition demonstrated a significant decrease in CARM1 accumulation and H3R17 methylation (Figure 6(c)).

## 4. Discussion

In the present work, we first identified DDR2 as a gene differentially expressed among hBM-MSCs, Y-hBM-MSCs, and hESCs using microarray and bioinformatics technologies. Then, the regulatory role of DDR2 in hBM-MSC senescence was confirmed by comparing the senescence phenotypes of early- and late-passage cells, as well as cells with siRNA-targeted inhibition of DDR2 expression. We also showed that CARM1 upregulates DDR2 expression via increased methylation of histone H3 at the latter's promoter region. Overexpression of either CARM1 or DDR2 significantly decreased the number of cells positive for the senescence marker *β*-Gal. Taken together, these results indicate that DDR2 might be a major contributor to the *in vitro* senescence resistance of hBM-MSCs and that CARM1-mediated histone arginine methylation at the DDR2 promoter region might be the key upstream regulatory mechanism.

MSC-based cell therapies have several drawbacks, including their dependence on *in vitro* expansion after initial isolation of a low quantity of hBM-MSCs from the bone marrow. Further, long-term *in vitro* culturing adversely affects hBM-MSCs by reducing their differentiation potential and promoting cellular senescence. Previous studies have suggested that hBM-MSC senescence is linked to specific epigenetic alterations [20, 22–24]. Progressive downregulation of TERT, Oct4, and Sox2 and upregulation of osteogenic genes such as Runx2 and ALP have been observed during MSC expansion, and these changes in gene expression were closely associated with epigenetic dysregulation of histone H3 acetylation in K9 and K14 [19]. Decreased expression of histone deacetylases (HDACs) was also observed in senescent MSCs, and treatment with HDAC inhibitors has been shown to induce cellular senescence [25, 26]. Additionally, HDACs may also play important roles in cellular senescence by regulating the expression of miRNAs targeting human high-mobility group protein A2 (HMGA2) through histone modifications. For example, HMGA2 protein overexpression has been shown to induce the PI3K/Akt/mTOR/p70S6K cascade, which in turn suppresses the expression of the senescence markers p16 (INK4A) and p21 (CIP1/WAF1) in human umbilical cord blood-derived multipotent stem cells (hUCB-MSCs) [27]. Additionally, our previous work showed that CARM1, by catalyzing histone H3 methylation at R17 and R26, plays an active role in hESC resistance to differentiation [21]. Therefore, we hypothesized that methylation of histone H3 at R17 might also contribute to the epigenetic regulation of certain senescence-associated genes in hBM-MSCs.

DDR2 is a tyrosine kinase collagen receptor expressed in mesenchymal tissues (e.g., smooth muscle cells, osteoblasts, and fibroblasts) [28]. DDR2 has been associated with cellular synthesis and the remodeling of fibrillar collagens (its ligand) within the extracellular matrix (ECM) [29] and is implicated in the expression and activation of matrix metalloproteinases [30–32]. DDR2 expression has also been shown to be increased during pathological scarring and epithelial-mesenchymal transition (EMT), a cellular transformation that mediates many stages of embryonic development and disease [33, 34]. For example, DDR2 activation is suggested to promote collagen production or remodeling in tumors [33]. A recent study suggested that DDR2 had relatively lower activation in old collagen compared to younger collagen, with a concomitantly high level of JAK2 and ERK1/2 phosphorylation and decreased expression of the cell cycle negative regulator p21^CIP1^. Inhibition of DDR2 kinase function also led to an increase in ERK1/2 phosphorylation and a decrease in p21^CIP1^ expression. Therefore, collagen aging promotes tumor cell proliferation by reducing the activation of DDR2 [35]. DDR2-deficient mice showed impaired chondrocyte proliferation that led to dwarfism [36], and DDR2 played an essential role in osteoblast differentiation and chondrocyte maturation via modulation of Runx2 activation [37]. More recently, DDR2 has been shown to be critical for the differentiation of marrow progenitor cells to osteoblasts while suppressing marrow adipogenesis [23]. As mentioned above, senescent MSCs were associated with decreased osteogenic but increased adipogenic potential [7, 8], which might be partially explained by the decreased level of DDR2 in those advanced cells. In this study, we found that after overexpressed CARM1, the level of DDR2 phosphorylation was increased. Therefore, the CARM1/DDR2 regulatory pathway might be a novel target for the molecular manipulation of hBM-MSC senescence.

Although inhibition of CARM1 or DDR2 expression both led to p16 upregulation, senescence phenotypes, and decreased cellular proliferation, the overexpression of CARM1 or DDR2 did not downregulate p16 or increase cellular proliferation. Previous studies have shown that both the p53/p21/RB and p16/RB axes are the key signaling pathways involved in the induction of cell senescence [38]. p16, p21, and p53 have also been demonstrated to be significantly upregulated in senescent hBM-MSCs [39]. In comparison, at the late stage of the hBM-MSC lifespan, significantly increased expression was observed in p16 but not p21 nor p53 [40]. Therefore, p16 might be the key marker for hBM-MSC senescence. The possible explanations for the consistent p16 expression level after overexpression of either CARM1 or DDR2 might be as follows: first, the senescence resistance effects of DDR2 might be dependent on other currently unidentified downstream factors. Therefore, overexpression of DDR2 alone is not sufficient to promote proliferation. Second, DDR2 and CARM1 might play different roles in different subpopulations of hBM-MSCs concerning the maintenance of proliferative capacity and senescence resistance. For instance, DDR2 might be vital to the majority of hBM-MSCs, which have a limited replication capacity and are more prone to *in vitro* replicative senescence. In comparison, in populations with high proliferative capacity (e.g., young adult-derived hBM-MSCs), DDR2 or CARM1 might have only limited effects. Therefore, further studies are needed to examine the expression profile of DDR2 and its related signaling pathway in a more homogeneous hBM-MSC population.

In conclusion, our results demonstrated that DDR2 expression is decreased in late-passage hBM-MSCs compared to their early-passage counterparts and that suppression of DDR2 by siRNA-mediated knockdown impaired self-renewal capacity and induced characteristic senescence features in early-passage hBM-MSCs. CARM1 is capable of binding to the DDR2 promoter region and preferentially methylated H3R17 in early-passage cells, and inhibition of CARM1-mediated histone arginine methylation was shown to decrease DDR2 expression and lead to senescence. Overexpression of CARM1 or DDR2 postponed senescence in late-passage cells. Therefore, the CARM1/DDR2 pathway might play a major role in the regulation of senescence in hBM-MSCs.

## Data Availability

(1) The data about real-time PCR, 5-ethynyl-2′-deoxyuridine (EdU) incorporation assays, *β*-Galactosidase staining, immunofluorescence staining, Western blot analysis, chromatin immunoprecipitation (ChIP) assay, growth curve, cell viability, and colony-forming unit fibroblast assay which were used to support the findings of this study are included within the article. (2) The data supporting this global DGE analysis are from previously reported studies and datasets, which have been cited. The processed data are available at GEO databases with the reference numbers GSM866168, GSM866169, GSM866170, GSM661349, GSM661350, and GSM661351.
